# An atypical RNA silencing suppression strategy provides a snapshot of the evolution of sweet potato-infecting potyviruses

**DOI:** 10.1038/s41598-018-34358-y

**Published:** 2018-10-29

**Authors:** Bernardo Rodamilans, Adrián Valli, Ares Mingot, David San León, Juan José López-Moya, Juan Antonio García

**Affiliations:** 1 Centro Nacional de Biotecnología CNB, CSIC, Madrid, Spain; 2 Center for Research in Agricultural Genomics CRAG, CSIC-IRTA-UAB-UB, Campus UAB Bellaterra, Cerdanyola del Vallès, Barcelona, Spain

## Abstract

Plant viruses usually encode proteins with RNA silencing suppression (RSS) activity to counteract plant defenses. In *Potyvirus*, the largest genus in the family *Potyviridae*, this role is taken over by the multifunctional HCPro, also involved in aphid transmission, polyprotein processing and virion formation. Recently, the large P1 of *Sweet potato feathery mottle virus* (SPFMV) was characterized finding an extra ORF produced after polymerase slippage, which originates the product P1N-PISPO. Transient expression assays showed that SPFMV P1 and P1N-PISPO presented RSS activity, while HCPro did not. In this work, we analyze possible differences between HCPro of SPFMV and other potyviruses, testing HCPro RSS activity in a transient expression assay, and using a *Plum pox virus*-based system to test the ability of SPFMV P1N-PISPO and HCPro to serve as RNA silencing suppressors in the context of a viral infection. Our results indicate that not only P1 and P1N-PISPO, but also HCPro display RSS activity when expressed in a suitable context, stressing the importance of the selected experimental system for testing anti-silencing capacity of proteins. The presence of multiple viral silencing suppressors in SPFMV adds complexity to an already intricate RSS system, and provides insight into the hypothetical evolution of sweet potato-infecting potyvirids.

## Introduction

*Potyviridae* is a family of plant infecting viruses, with members presenting a positive-sense single-stranded RNA genome of around 10 Kb^1,2^. It currently comprises nine monopartite and one bipartite genera^3^. In the monopartite genera, most of the proteins are expressed from a single open reading frame (ORF) that produces a polyprotein with at least ten different products. The majority of these products are separated by the *cis*- and *trans*- action of the NIa protease^4^, with additional contribution of other virus-encoded endopeptidases (reviewed by^1^). Recently, extra ORFs were described as a consequence of polymerase slippage events at a conserved GA_6_ motif in the central region of P3^5–7^. Hence, addition or deletion of a single A originates the frameshift products P3N-PIPO or P3N-ALT, respectively. Both proteins are involved in viral movement^8–10^.

*Potyvirus* is the largest genus by far inside the family with ca. 200 members^1,2^, and viruses within this group follow a conserved genomic organization by encoding two proteases at the 5′ end: P1 and HCPro. P1 is a serine protease with autocatalytic activity dependent on plant co-factor(s)^11–13^. Classified as a Type A P1 protein, it is the most variable potyviral factor in length and amino acid sequence^14,15^. It is a non-essential factor involved in host range definition^15–18^, whose protease activity appears to modulate viral replication facilitating the escape from host defenses^19–21^. Additional P1 functions, including preferential translation of viral mRNAs, have been suggested^20,22,23^.

HCPro, instead, is an autocatalytic cysteine protease whose activity does not appear to rely on specific host factors^24,25^. It is an essential protein for which diverse roles have been described, with the suppression of antiviral silencing being the most prominent^26^. Whereas HCPro is the canonical RNA silencing suppressor of viruses of the genus *Potyvirus*, members of other genera of the family *Potyviridae* encode HCPros that have been described as defective in this essential function. In these viruses, silencing is counteracted by a second group of P1 proteins (Type B)^21,27–32^.

A number of viruses of the genus *Potyvirus* infects sweet potato and present an enlarged P1^33^. Of these, the most relevant is *Sweet potato feathery mottle virus* (SPFMV), which causes the devastating sweet potato viral disease (SPVD) in mixed infections with the crinivirus *Sweet potato chlorotic stunt virus* (SPCSV)^34,35^. The P1 of SPFMV has been recently characterized^36,37^. This protein carries a GA_6_ motif similar to the one observed in P3, which generates, through an equivalent polymerase slippage event, an extra ORF that encodes the product P1N-PISPO. Both, P1 and P1N-PISPO, have been detected in natural viral infections, and both display RNA silencing suppression (RSS) activity in transient expression assays mediated by agroinfiltration. The RSS activity of P1 appears to work only at local level^37^, while the one of P1N-PISPO, related to WG/GW motifs and AGO binding, seems to prevent local silencing and short-distance movement of the silencing signal^36,37^. Interestingly, agroinfiltration assays carried out by two research groups in independent parallel experiments failed to show RSS activity for the HCPro of SPFMV^36,37^.

This study analyze the RSS activity of proteins encoded in the 5′ region of the SPFMV genome in the context of a viral infection, paying special attention to possible anomalies that seems to disrupt the expected canonical activity of SPFMV HCPro.

## Results

### Analysis of SPFMV HCPro sequence does not justify a lack of RSS activity

Whereas the absence of RSS activity is the rule for the HCPro from viruses of the *Ipomovirus, Tritimovirus* and *Poacevirus* genera, the inability of SPFMV HCPro to suppress silencing in a typical agroinfiltration system^36,37^ is a striking exception among potyviruses.

To search for differences at the level of the primary structure that could give an explanation for the exceptional behavior of SPFMV HCPro, the amino acid sequence of this protein was compared to the HCPro of 86 potyviruses (Supplementary Table 1). The average size of HCPros was 51.8KDa ± SD 1.17. Only two outliers were found, *Onion yellow dwarf virus* (OYDV) that bears an HCPro of 41.8KDa, and *Donkey orchid virus A* (DOVA) with an HCPro of 63.4KDa. This size consistency is remarkable considering that the N-terminal region, except for the KITC motif involved in aphid transmission^38,39^, is not conserved in terms of amino acid sequence and is not essential for the infection of some potyviruses^39,40^.

**Figure 1:**
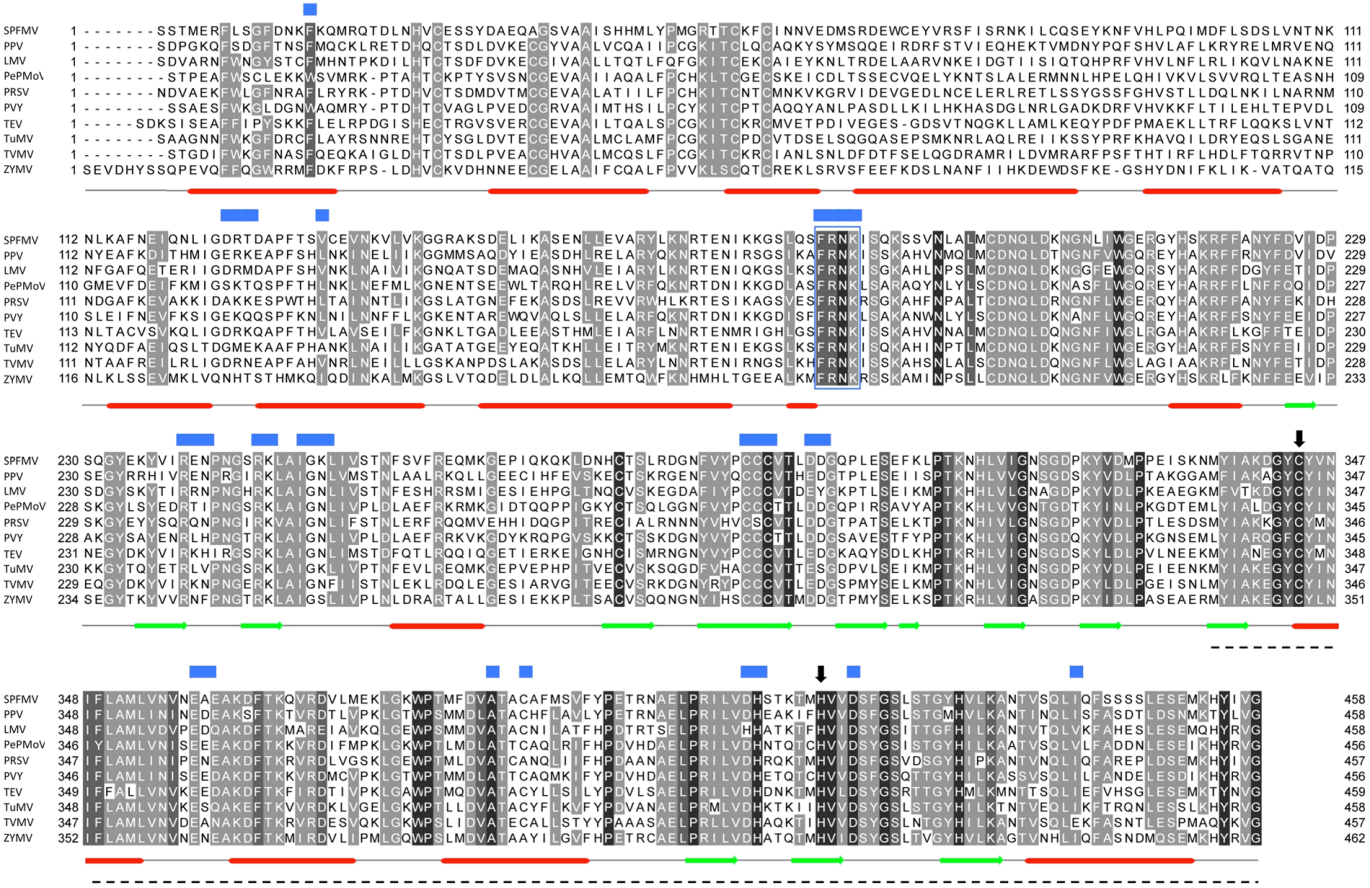
Sequence alignment of potyviral HCPro. Amino acid sequences of HCPro from 85 different potyvirus species have been used for the alignment (see Supplementary Fig. S1). In black are marked positions in which amino acids are identical. Dark grey mark positions in which amino acids are not identical, but are conserved^15^ in all 85 species. Pale grey mark positions in which amino acids are conserved at least in 70 viral species (~80% of the total). Ten representative species have been selected for easier visualization. N-terminal and C-terminal ends of each fragment are indicated. Dashes represent gaps. Blue squares mark the residues described as relevant for RSS^26^. The FRNK motif is also marked inside a blue square. Black arrows indicate the two catalytic residues of the cysteine protease domain. Secondary structure prediction, performed with Jpred is indicated below the sequences. Dashed line indicate the part of TuMV HCPro that was solved by X-ray crystallography.

The alignment of 85 potyviral species (HCPro outliers were excluded) allowed us to create a reliable map of conserved amino acids (Fig. 1, Supplementary Fig. S1). As anticipated, the protease C-terminal region is the one presenting the highest conservation, while the N-terminal part is less conserved across species. This is also true for the sweet potato-infecting potyviruses that do not show any differences in this regard to the rest of the species in the genus. In Fig. 1, we marked the motifs described to be relevant for HCPro-mediated RSS activity^26^. The FRNK motif, located in the middle region of the protein is the best characterized motif described as relevant for RSS^41^, and the alignment of potyviral species shows that SPFMV and the rest of the sweet potato-infecting potyviruses conserve not only this motif, but also the rest of the amino acids relevant for this activity (Fig. 1, Supplementary Fig. S1). Our comparison indicates that there are not remarkable differences in terms of primary structure or motif conservation that could explain why SPFMV HCPro lacks RSS activity.

### SPFMV HCPro, even preceded of P1, does not present RSS activity in transient expression assays

**Figure 2:**
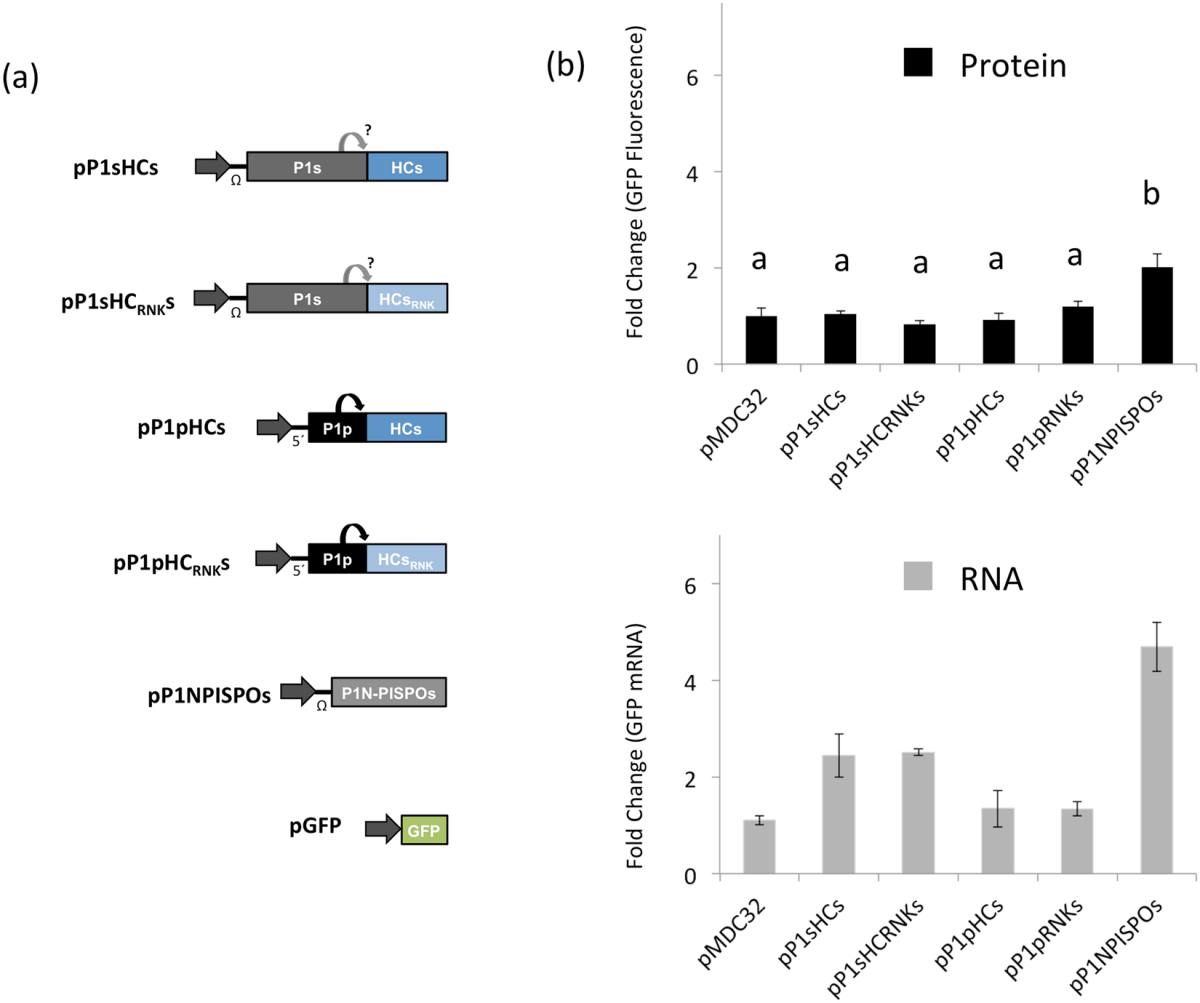
Transient agroinfiltration assay to test RSS activity of SPFMV HCPro. (**a**) Schematic representation of transient expression plasmids. Letters after each protein indicate viral species (p: PPV, s: SPFMV). Grey horizontal arrows represent the 35S promoter. Round arrows mark autocatalytic cleavage. (**b**) *N. benthamiana* plants were co-agroinfiltrated with GFP and the designated construct. In the upper panel, discs from agroinfiltrated leaves were collected at 6 dpa and GFP fluorescence was quantified in a 96-well plate reader. Relative GFP signal intensities are indicated using mean value of the negative control, pMDC32, as 1. Bars show mean ± SD (*n* = 4) and letters indicate *p* < 0.05 in one-way ANOVA and Tukey´s post hoc test. In the lower panel, discs used for GFP fluorescence were collected and RNA was extracted to perform RT-qPCR analysis. Relative accumulation of GFP mRNAs measured by specific RT-qPCR and normalized against the mean value of the negative control, pMDC32, is shown. Bars show mean ± SD (*n* = 2). Kruskal-Wallis test showed significant differences (p < 0.05), but none of the pairwise comparisons were significant.

Given the apparent inconsistency between the lack of RSS activity of SPFMV HCPro in classic co-agroinfiltration assays previously published^36,37^ and our primary sequence analysis, we decided to further investigate the discrepancies by performing another transient expression assay in *N. benthamiana* plants using a new set of constructs and GFP as inducer and reporter of RNA silencing (Fig. 2a). SPFMV HCPro was not expressed alone as in the previous works, but preceded by SPFMV P1 (pP1sHCs) since it was reported that P1 might enhance HCPro RSS activity^27,42,43^. This construct, however, presented two possible problems: first, SPFMV P1 can display RSS activity on its own^37^, which might hinder the interpretation of the results; and second, the autocatalytic activity of SPFMV P1 in *N. benthamiana*, which is required to separate itself from the downstream proteins, might not work properly, so that the production of a free (and possibly active) HCPro is not warrantied^19,21^. To overcome these issues, we (i) included as a control a construct that expresses P1 along with a mutated version of HCPro (RK846,848AA) that is expected to abolish HCPro RSS activity if any (pP1sHC_RNK_s)^41,44,45^; and we (ii) included two equivalent constructs in which SPFMV P1 was replaced by PPV P1, which is fully active as a self-protease in *N. benthamiana* and seems to enhance the RSS activity of downstream HCPro^27^ (pP1pHCs and pP1pHC_RNK_s). SPFMV P1N-PISPO (pP1N-PISPOs) was used as positive control (Fig. 2a).

The transient expression assay was performed in *N. benthamiana* and after 6 days post-agroinfiltration (dpa) GFP fluorescence was measured in little discs coming from agroinfiltrated leaf patches (Fig. 2b, upper panel). Discs were further used for RT-qPCR analysis (Fig. 2b, lower panel). Emission of fluorescence indicated there were no significant differences between co-expressing GFP with an empty vector or with any of the constructs carrying SPFMV HCPro (pP1sHCs, pP1sHC_RNK_s, pP1pHCs or pP1pHC_RNK_s). Co-expression of the reporter with pP1N-PISPOs doubled the amount of GFP fluorescence compared to the control (Fig. 2b, upper panel). On the other hand, RT-qPCR analysis showed noticeable differences in GFP mRNA levels in samples co-agroinfiltrated with pP1sHCs compared to the empty vector. Similar increase (around two folds) was also detected with pP1sHC_RNK_s, which suggest that, as reported^37^, the protein responsible for this activity is P1 from SPFMV and not HCPro. In line with this conclusion, samples co-agroinfiltrated with plasmids carrying PPV P1 instead of SPFMV P1 (pP1pHCs and pP1pHC_RNK_s) did not show appreciable differences in GFP mRNA levels with respect to co-agroinfiltration of the GFP reporter construct with an empty vector. Plants agroinfiltrated with pP1N-PISPOs showed an increase in GFP mRNA levels of approximately four times compared to the empty vector (Fig. 2b, lower panel). Altogether these results were consistent with those obtained in previous reports^36,37^, confirming that SPFMV HCPro does not display RSS activity in transient expression tests.

### SPFMV P1N-PISPO can replace PPV HCPro to support systemic infection in N. benthamiana

It was reported that several heterologous RNA silencing suppressors can replace PPV HCPro and rescue an otherwise defective virus, allowing PPV infection in *N. benthamiana* plants^46^. This showed how relevant is the role of HCPro as an RNA silencing suppressor protein and, in addition, represented an adequate method for testing possible RNA silencing suppressors in the context of a viral infection.

**Figure 3:**
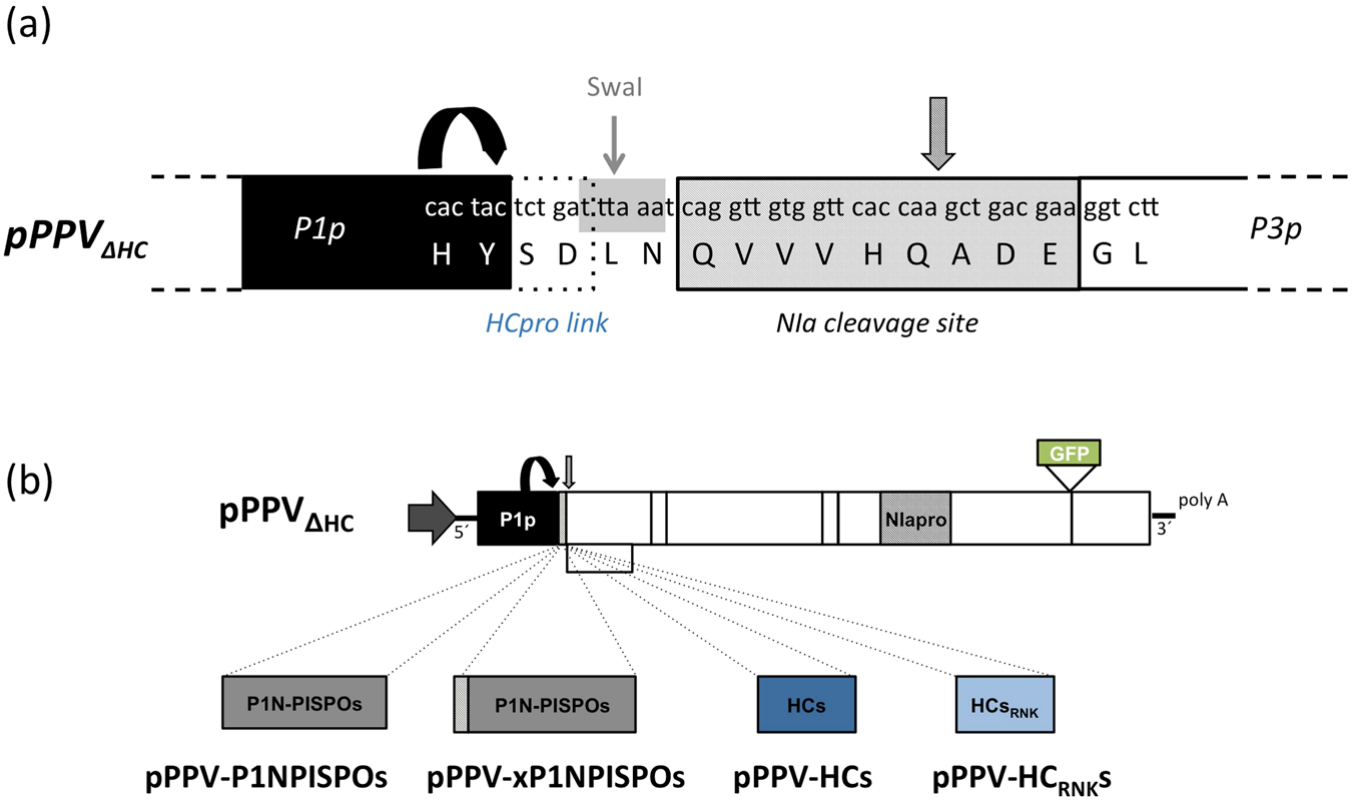
Schematic representation of PPV-derived viral constructs. (**a**) Detailed view of the cloning site of the modified plasmid pPPV_∆HC_. HCPro linker included to facilitate PPV P1 autocleavage (round arrow) is marked with a dotted box. SwaI site engineered for cloning of the desired genes is marked in grey. NIa cleavage site is indicated with a striped box and a striped vertical arrow. (**b**) Full-length cDNA clones. Grey horizontal arrows represent the 35S promoter. Round arrow marks the autocatalytic cleavage of PPV P1. NIa cleavage site is marked with a striped box and a striped vertical arrow. The GFP coding sequence inserted between NIb and CP cistrons is marked as a green box.

Thus, a previously described PPV vector (pPPV in this work)^47^ was modified to be used as cloning template for the replacement of PPV HCPro by other proteins to be tested (pPPV_ΔHC_). The coding sequence of HCPro was removed and a sequence corresponding to the target site for the viral protease NIa was introduced. The sequence corresponding to the first two amino acids of HCPro (Ser and Asp) was left after P1 to facilitate its autocleavage (Fig. 3a). To test the validity of the newly designed plasmid and, at the same time, assess whether SPFMV P1N-PISPO, which displayed weak RSS activity in transient expression tests, was able to rescue a PPV virus that carries no HCPro, we prepared two viral constructs: one carrying the sequence of SPFMV P1N-PISPO (pPPV-P1NPISPOs) and another one with an extra NIa cleavage site at the N-terminus of P1N-PISPO to ensure the release of PPV P1 from the viral polyprotein (pPPV-xP1NPISPOs) (Fig. 3b). Four plants of *N. benthamiana* were agroinoculated with each of the modified PPV clones. Two of these plants were also co-infiltrated with agrobacteria carrying an expression plasmid for the known RNA silencing suppressor p19 from *Tomato bushy stunt virus*. In this way, we could verify at the inoculated leaves that GFP was produced, indicating that all viral cistrons were correctly in frame after the cloning while also ensuring the production of the corresponding RNA silencing suppressor. pPPV and pPPV_∆HC_ were used as positive and negative controls, respectively.

**Figure 4:**
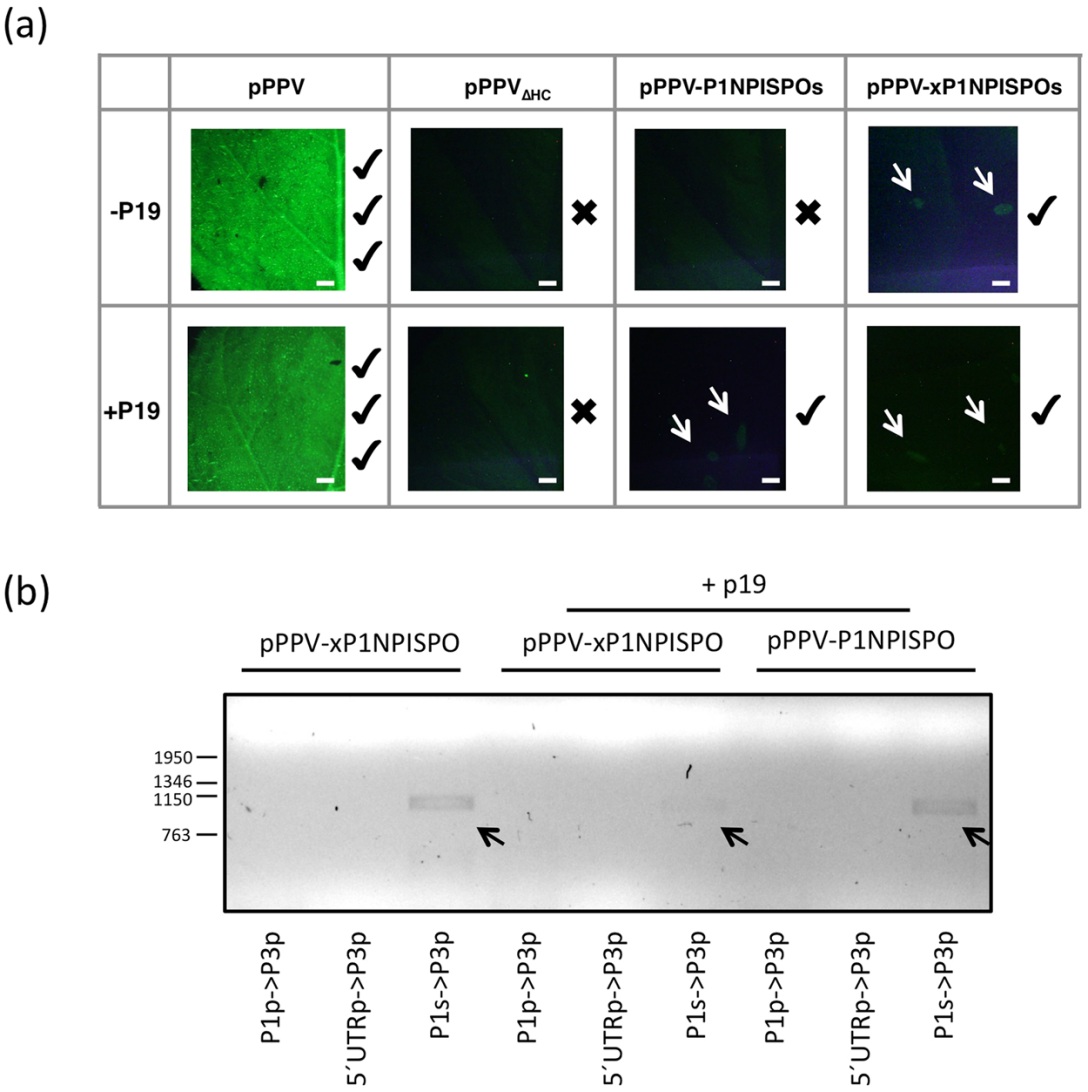
Infection of *N. benthamiana* plants with a chimeric PPV carrying the coding sequence of SPFMV P1N-PISPO instead of the PPV HCPro cistron. (**a**) Pictures of agroinfiltrated leaves of *N. benthamiana* plants taken under an epifluorescence microscope at 13 dpa; scale bars, 2 mm. Intensity of pPPV images was reduced 40% to avoid saturation. Checkmarks and crosses next to the pictures indicate the presence or absence, respectively, of GFP foci. Foci in pPPV-P1NPISPOs and pPPV-xP1NPISPOs are marked with white arrows. (**b**) PCR amplification after reverse transcription using three different pairs of primers for specific amplification of pPPV (P1p- >P3p), pPPV_∆HC_ (5´UTRp- >P3p) and pPPV-P1NPISPOs/ pPPV-xP1NPISPOs (P1s- >P3p). The size (in bp) of DNA markers run in the same gel is shown to the left of the panel. Bands that were extracted and send for sequencing are marked with black arrows.

Presence of viral accumulation was assessed first by checking GFP fluorescence using a stereoscope microscope at 8 dpa. All plants co-agroinfiltrated with p19 displayed GFP fluorescence at the inoculated leaves. However, at the upper non-inoculated tissue, GFP could only be observed in plants treated with pPPV (not shown). At 12 dpa very small foci could be detected in one of the upper leaves from one of the plants co-agroinfiltrated with p19 and pPPV-P1NPISPOs, and from two of the plants agroinfiltrated with pPPV-xP1NPISPOs (with and without p19) (Fig. 4a, white arrows). To confirm the infection and to verify that the observed foci contained virus carrying SPFMV P1N-PISPO, the green fluorescent foci were collected, RNA was extracted from this tissue and viral identification by RT-PCR was performed with three different set of primers amplifying regions from pPPV, pPPV_∆H_ or pPPV-P1NPISPOs/pPPVxP1NPISPOs. In all three cases bands were obtained only with the primers that amplify the sequence from SPFMV P1N-PISPO, covering a region of 1009 bp (Fig. 4b, black arrows). Sanger sequencing of these bands confirmed the presence of SPFMV P1N-PISPO in these samples. No extra foci appeared at later times of infection. A confirmatory agroinoculation experiment was repeated in *N. benthamiana* plants using pPPV-xP1NPISPOs and the same controls as before. Foci were found in three out of five plants inoculated with pPPV-xP1NPISPOs, at 12 dpi, corroborating the previous results (data not shown).

### SPFMV HCPro can replace HCPro from PPV with high efficiency, allowing systemic infection in N. benthamiana plants

**Figure 5:**
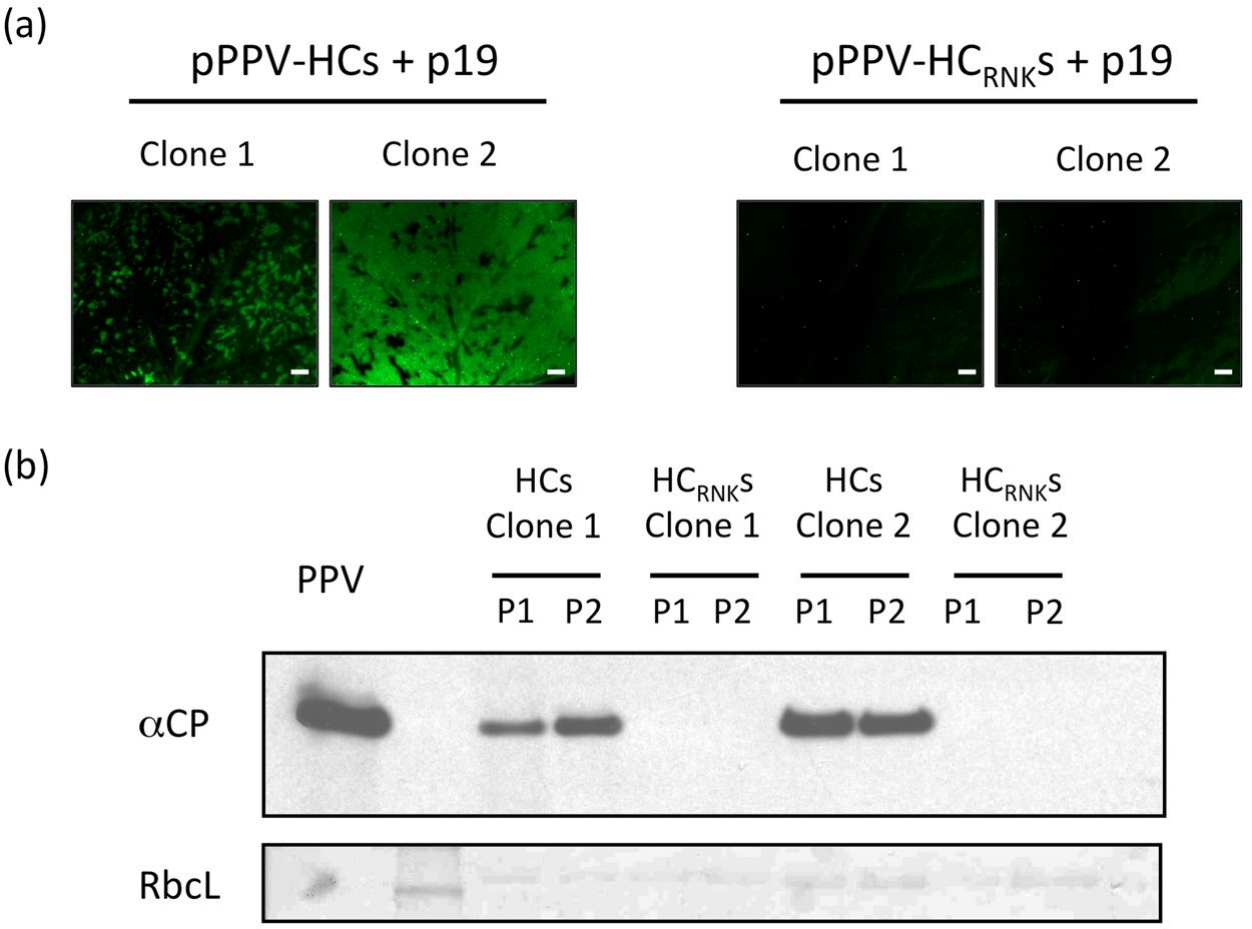
Infection of *N. benthamiana* plants with a chimeric PPV carrying the coding sequence of SPFMV HCPro instead of the PPV HCPro cistron. (**a**) Pictures of agroinfiltrated leaves of *N. benthamiana* plants taken under an epifluorescence microscope at 11 dpa; scale bars, 2 mm. (**b**) Viral accumulation was assessed by anti-CP inmunoblot assay. Each lane corresponds to a pool of two upper non-inoculated leaves from a single plant; RbcL, Ponceau red-stained blots showing the large subunit of Rubisco protein as loading control.

As anticipated from previous reports^36,37^ and confirmed by our results above, SPFMV HCPro lacks RSS activity in classic co-agroinfiltration assays (Fig. 2). However, the putative anti-silencing activity might need the context of a viral infection to be revealed. Taking advantage of the modified PPV plasmid, pPPV_∆HC_, SPFMV HCPro and a theoretically defective mutant in RSS activity (HC_RNK_s) were inserted in place of PPV HCPro to generate the chimeric viruses pPPV-HCs and pPPV-HC_RNK_s, respectively (Fig. 3b). These viral constructs were used to agroinoculate *N. benthamiana* plants. As in the previous experiment, viruses were expressed along with p19 to verify at the inoculated leaves whether GFP as well as the corresponding RNA silencing suppressors were produced. Two independent clones were used in each case and two plants were inoculated with each clone. At 8 dpa all plants showed GFP fluorescence at the inoculated leaves (not shown). At 11 dpa all four plants inoculated with pPPV-HCs showed external disease symptoms in the upper non-inoculated leaves typical of PPV infection with some chlorosis and leaf curling, whereas no symptoms were detected in plants treated with pPPV-HC_RNK_s (not shown). Observation of upper non-inoculated leaves under UV light in the stereomicroscope revealed the presence of GFP only in plants inoculated with pPPV-HCs (Fig. 5a). The presence of virus in this tissue was confirmed by anti-CP immunoblot analysis. Plants inoculated with pPPV-HC_RNK_s did not show CP accumulation (Fig. 5b). The identity of pPPV-HCs chimera was verified by RT-PCR followed by Sanger sequencing of the generated product (not shown). A second experiment was performed (Fig. S2) confirming our above-explained observations.

## Discussion

RNA silencing suppressors are essential part of viral counterdefense mechanisms during plant-pathogen interaction. Most, if not all, viruses encode at least one protein with RSS activity^48–50^. SPFMV encodes two P1 gene-related proteins: P1 itself, produced by the main ORF, and P1N-PISPO, produced by an RNA variant derived from polymerase slippage. Both proteins have been reported to present RSS activity in co-agroinfiltration tests. In contrast, SPFMV HCPro showed no activity in the same assays^36,37^. Thus, the potyvirus SPFMV appeared to resemble ipomoviruses, tritimoviruses and poaceviruses, whose RSS activity depends on a Type B P1 protein rather than on HCPro^27,29–32^. In addition, SPFMV P1 presents strong similarities with the N-terminal part of P1 of the ipomovirus *Sweet potato mild mottle virus* (SPMMV), which bears WG/GW motifs for AGO binding responsible for its RSS activity^15,29,51^, reinforcing the notion that SPFMV HCPro might be behaving as its RSS-deficient ipomoviral counterpart.

Our initial alignment of potyviral HCPros (Fig. 1, Supplementary Fig. S1) was intended to find differences between SPFMV HCPro and other potyviral HCPros that could explain its apparent lack of RSS activity. However, the comparison did not show any significant changes in size or in presence and position of any of the motifs described as critical for such a protein role, including the well-characterized FRNK motif^26^. This apparent inconsistency between *in silico* analysis and previously reported experimental data led us to perform a series of experiments to further test the validity of the original findings. In this way, we completed new co-agroinfiltration tests and confirmed that SPFMV HCPro did not work as an RNA silencing suppressor in the classical transient expression system (Fig. 2). Several reasons might account for this lack of RSS activity: a failure in expression/stability of SPFMV HCPro; a reduced RSS activity that makes SPFMV HCPro unable to counteract the degradation of the constitutively expressed GFP mRNA; a genuine deficiency of RSS activity of SPFMV HCPro; and others.

To explore some of these possibilities, SPFMV HCPro RSS activity was tested in a different expression system, such as the one based on pPPV_∆HC_ that allows testing suppressors of RNA silencing in the context of a viral infection. In this case, SPFMV HCPro-carrying PPV was infectious, moved unrestricted through the younger leaves and accumulated to high amounts, as shown by western blot. *Cis*-complementation of the defective pPPV_∆HC_ by SPFMV HCPro might be due to functions of this protein, not related to RSS, which would be enough to support viral infection. However, this possibility is unlikely, as the PPV chimerical virus that carries the SPFMV HCPro with a mutation in the well known antisilencing-related FRNK motif^41^ (HC_RNK_) was not able to spread towards upper non-inoculated leaves (Figs 5 and S2). Hence, our results strongly suggest that this protein is displaying RSS activity during viral infection.

The inconsistency between results from transient expression and viral infection assays reported here highlights the relevance of the experimental system of choice when testing the RSS activity of a given protein, and warns about conclusions drawn out of negative outcomes. Test system incompatibilities have been described before, such in the case of CTV CP, which only showed consistent RSS activity in a transgenic plant system^52^, but they were not anticipated in the case of a potyviral HCPro, considering that all other HCPros from several potyviral species reported so far had shown strong RSS activity in transient co-agroinfiltration assays (see, for instance,^27,31,37,41,53–56^).

The highly efficient systemic infection of the PPV chimera expressing SPFMV HCPro (Fig. 5) contrasts with the very scarce infection of the chimera expressing SPFMV P1N-PISPO (Fig. 4) and suggests that, as it is typical for potyviral infections, HCPro is the main contributor to silencing suppression in SPFMV-infected plants. HCPro has been shown to be the factor responsible for the synergistic transactivation of heterologous viruses, which has been associated with its RSS activity^57,58^. The fact that SPFMV HCPro facilitates systemic spread of PVX in sweet potato^59^ further supports the functional relevance of the RSS activity of HCPro in SPFMV infection.

RNA silencing suppressors are diverse and likely evolved recently and independently from previously existing viral proteins^48^. Viruses normally encode a single protein with RSS activity that is tightly regulated to avoid hyper- or hyposuppression^20,60,61^. The presence of two RNA silencing suppressors in the same virus, however, is not strange and even some plant viruses, like *Citrus tristeza virus* (CTV)^52^ and *Tomato chlorosis virus*^62^ of the *Closteroviridae*, and *Broad bean wilt virus 2* of the *Secoviridae*^63^, present more than two RNA silencing suppressors. In the *Potyviridae*, in addition to the canonical HCPro, a second multifunctional protein, VPg, has been reported to have RSS activity^64–66^. This activity has been demonstrated for PVA and TuMV, but was suggested to be an auxiliary function not absolutely required for infection in *N. benthamiana*^65^. The notion of several RNA silencing suppressors present in the same virus can relate to a synergistic effect in which the different suppressors block independent components of the defense system at the intra- and intercellular level. This was suggested for CTV and for *Potato virus M*^52,67^, and might also be the case for SPFMV in which P1, P1N-PISPO and HCPro seem to have different modes of action^37^. Nonetheless, it is intriguing why only potyviruses infecting sweet potato would need this combination of silencing suppressors. The reduction of polymerase slippage rate, and therefore P1N-PISPO production, in SPFMV during mixed infections with SPCSV, which in nature cause SPVD^34–36^ suggests that SPFMV RSS activities can be further modulated by external factors. This adds complexity to an already intricate scenario and reinforces the necessity of understanding the various interactions in order to confront devastating diseases such as SPVD.

On the other hand, the peculiar RSS pattern observed in the SPFMV group of potyviruses might not be related to specific requirements of these viruses and/or sweet potato plants, but to the complex evolutionary history of the family *Potyviridae* (discussed in^15,29^). The strong sequence similarity between the potyvirus SPFMV and the ipomovirus SPMMV suggests that these viruses could be linked by an intergenera recombination event^15^. In this scenario, we could envisage a major ancestor of SPMMV carrying P1 and P1N-PISPO with WG/GW-based RSS activity and an RSS-deficient HCPro. SPMMV would directly derive from this ancestor by losing the PISPO ORF, while SPFMV would be the result of the incorporation of the N-terminal part of P1 of this ancestor, including the slippage motif, to the P1 of a potyvirus with a functional HCPro. The following observations support this view: i) in spite of the monophyletic origin of PISPO-containing viruses, PISPO is rather poorly conserved^37^; ii) SPFMV P1 RSS activity is very weak (Fig. 3), but introduction of two point mutations that recreate a WG/GW motif, resembling SPMMV P1, significantly improved it^37^, suggesting that SPFMV P1 already suffered an RSS rearrangement; and iii) instead of an experimental artifact, the lack of RSS activity of SPFMV HCPro in the agroinfiltration system might be interpreted as the result of a divergent process suffered by this particular HCPro due to the incorporation of other sources of RRS activity.

In any case, the present work advances in the understanding of RNA silencing suppressors in the *Potyviridae* family by adding clues to possible evolutionary routes and emphasizing the significance of the experimental system when testing complex activities such as RSS.

## Methods

### Alignment of Potyviral HC amino acid sequences

Multiple sequence alignment was performed initially by ClustalW using MegAlign®. Version 8.0.2. DNASTAR. Madison, WI. Analysis was refined by manual editing using Jalview software^68^. Viruses used in the alignment are detailed in Supplementary Table 1.

### Plasmids

pGW702Ω-P1NPISPO (pP1NPISPOs) was reported^36^. pGFP and pBIN61:p19 were kindly provided by D. Baulcombe (University of Cambridge, United Kingdom). The rest of the plasmids were generated using standard molecular cloning procedures. PCR reactions were performed with Phusion High-Fidelity DNA Polymerase (New England BioLabs). Primers were synthesized by Sigma-Aldrich and sequencing of all products was carried out by Macrogen. Primers are listed in Supplementary Table 2. Restriction enzymes and T4-DNA ligase were purchased to Thermo Fisher Scientific and New England BioLabs.

*pPPV*_*∆HC*_. Two DNA fragments were amplified from the reported pBINPPV-NK-GFP plasmid (pPPV)^47^ using primers 2672/2673 and 2674/2675, respectively. DNA fragments were joined together by overlapping PCR using primers 2672/2675 and the resulting product was cloned into pUC19, previously digested with SmaI. The PPV fragment of the pUC19-derived intermediate clone was excised by digestion with XmaI, and ligated into pPPV digested with the same enzyme and dephosphorylated.

*pPPV-P1NPISPOs, pPPV-xP1NPISPOs, pPPV-HCs, pPPV-HC*_*RNK*_*s*. P1NPISPOs and xP1NPISPOs sequences were amplified by PCR using oligonucleotides 2676/2678 and 2709/2678, respectively, as primers, and pP1NPISPOs as template. HCs and HC_RNK_s sequences were amplified by PCR using primers 2679/2680 and pP1sHCs and pP1sHC_RNK_s, respectively, as templates. All PCR products were ligated to pPPV_∆HC_, digested with SwaI, including SwaI in the reaction mix to reduce background colonies^69^.

*pP1sHCs, pP1sHC*_*RNK*_*s*. The plasmid pGW702Ω-P1HCspfmv (pP1sHCs) was obtained following procedures already described^36^. Shortly, the two P1 and HC viral gene products were RT-PCR amplified with primers P1sF/HCsR from a total nucleic acid sample extracted from virus-infected plant tissues. To obtain P1sHC_RNK_s PCR product, initial PCRs were performed using as template pP1sHCs and using as primers P1sF/HCsmutR and HCsmutF/P1sR followed by an overlapping PCR with primers P1sF/HCsR. PCR products were cloned directionally into pENTR/D-TOPO before being mobilized into pGW702Ω using LR mixture (Thermo Fisher Scientific).

*pP1pHCs, pP1pHC*_*RNK*_*s*. P1pHCs and P1pHC_RNK_s sequences were amplified using primers 697/3054 and templates pPPV-HCs and pPPV-HC_RNK_s, respectively. PCR products carrying the gateway recombination sites were introduced first in pDONR207 and then in pMDC32 using BP and LR mixtures, respectively (Thermo Fisher Scientific).

### Agroinfiltration and fluorescence measurement

*N. benthamiana* plants were grown in a greenhouse maintained at a 16 h light/8 h dark photoperiod and a temperature range of 19-23 °C. Plants were infiltrated as described^27^, with *A. tumefaciens* strain C58C1-313^70^ carrying the indicated binary plasmid and using an OD_600_ of 0.5 for each construct. Two independent clones for each construct were used and two plants were agroinfiltrated with each clone. A stock of the cultures in 20% glycerol was saved at −80 °C and used in further experiments.

For the transient expression experiments, GFP fluorescence intensity quantification was carried out placing individual 5.0 mm-diameter leaf discs in a black 96-well plate (Nunc) filled with 50 µl water/well and acquiring the signal in a monochromator-based plate reader (Infinite M200, Tecan Group)^71^. Four discs per plant were collected. Average measurements of each plant were used for the analysis of variance after checking normality and homoscedasticity (*n* = 4, *p* < 0.05) (one-way ANOVA), followed by Tukey´s post hoc test.

For plants agroinoculated with PPV-derived constructs, analysis of infection of inoculated and upper leaves was carried out under an epifluorescesce microscope (MZ FLIII, GFP3 filter; Leica). Photos were taken with an Olympus DP70 digital camera at the indicated times.

### RT-PCR and RT-qPCR analyses

For the transient expression experiments, the same discs used for the fluorescence analysis were pulled and used for total RNA extraction using TRIzol reagent (Thermo Fisher Scientific). cDNA was prepared using Superscript III (Thermo Fisher Scientific). Technical triplicate qPCR reactions were prepared using HOT FIREPol EvaGreen qPCR Mix Plus (Solis BioDyne) in 96-well optical plates and run in a 7500HT Fast Real-Time PCR System (Applied Biosystems). Primer pairs 3227/3228^36^ and 2806/2807^72^ were used for GFP and PP2A amplification, respectively. Relative quantification was performed as described^73^. Kruskal-Wallis test was performed (*n* = 2, *p* < 0.05) followed by pairwise comparisons.

For plants agroinfiltrated with PPV-derived constructs, collected foci (13 dpi) from pPPV-P1NPISPOs- and pPPV-xP1NPISPOs-inoculated plants were used for total RNA extraction using TRIzol (Thermo Fisher Scientific). RT-PCR was performed in a single step using TITAN enzyme mix (Roche) with primer pairs 630/441, 270/441 and 2508/441 for the amplification of pPPV, pPPV_∆H_ and pPPV-P1NPISPO/ pPPVxP1NPISPOs, respectively. PCR products were sequenced. Tissue from pPPV-HCs- and pPPV-HC_RNK_s-inoculated plants was collected at 11 or 14 dpa (experiment 1 and 2, respectively) and used for Western blot analysis as well as RNA extraction using TRIzol (Thermo Fisher Scientific). RT-PCR was performed in a single step using TITAN enzyme mix (Roche) with primers 90 and 317 and the PCR product was sequenced. Sequencing of the PCR-amplified products was conducted by Macrogen.

### Western blot analysis

Plant tissue was ground in a mortar under liquid nitrogen. Crude extracts were prepared by homogenization in cracking buffer (125 mM Tris-HCl, 2% SDS, 6 M urea, 5% β-mercaptoethanol, 10% glycerol, 0.05% bromophenol blue, pH 7.5) using a mass:volume ratio of 1:2. Proteins were separated by 12% glycine-SDS-PAGE and electroblotted onto nitrocellulose membranes as reported^27^. Anti-PPV CP was used as primary antibody for protein detection and horseradish peroxidase conjugated goat anti-rabbit IgG (Jackson) was used as secondary antibody. Immunostained proteins were visualized by enhanced chemiluminescence detection with Clarity Western ECL Substrate (BioRad).

## Electronic supplementary material

Supplementary File
