# Supplementary material for: An atypical RNA silencing suppression strategy provides a snapshot of the evolution of sweet potato-infecting potyviruses
Source: Sci Rep. 2018 Oct 29;8:15937. doi: 10.1038/s41598-018-34358-y (PMC6206096; doi:10.1038/s41598-018-34358-y)

Supplementary Information

An atypical RNA silencing suppression strategy provides a snapshot of the evolution of sweet potato-infecting potyviruses

Bernardo Rodamilans[1*], Adrián Valli[1], Ares Mingot[2], David San León[1], Juan José López-Moya[2], Juan Antonio García[1]

[1] Centro Nacional de Biotecnología CNB, CSIC, Madrid, Spain

[2] Center for Research in Agricultural Genomics CRAG, CSIC-IRTA-UAB-UB, Campus UAB Bellaterra, Cerdanyola del Vallès, Barcelona, Spain

* Corresponding author: Bernardo Rodamilans Ramos. email: brodamilans@cnb.csic.es

This file includes:

Supplementary Table 1

Supplementary Table 2

Figure S1

Figure S2

Full-length gel from Figure 4

Full-length blots from Figure 5

Full-length gel from Figure S2

Supplementary Table 1. List of virus species used for HCPro sequence analysis

Acronym	Species	Accession
AMoV	*Arracacha mottle virus*	DQ925486
ApVY	*Apium virus Y*	HM363516
AWMV	*Algerian watermelon mosaic virus*	EU410442
BarMV	*Basella rugose mosaic virus*	DQ821938
BBrMV	*Banana bract mosaic virus*	DQ851496
BCMV	*Bean common mosaic virus*	AY863025
BCNMV	*Bean common necrosis mosaic virus*	AY282577
BiMoV	*Bidens mottle virus*	AF538686
BSVA	*Blue squill virus A*	JQ807999
BTBLV	*Brugmansia mosaic virus*	JX867236
BtMV	*Beet mosaic virus*	AY206394
BYMV	*Bean yellow mosaic virus*	AB079886
CABMV	*Cowpea aphid-borne mosaic virus*	HQ880242
CDV	*Colombian datura virus*	JQ801448
CeMV	*Celery mosaic virus*	HQ676607
ChiRSV	*Chilli ringspot virus*	JQ234922
ChiVMV	*Chilli veinal mottle virus*	AJ237843
CLLV	*Calla lily latent virus*	EF105297
ClYVV	*Clover yellow vein virus*	AB011819
CSV	*Cocksfoot streak virus*	EU119422
DapMV	*Daphne mosaic virus*	DQ299908
DOVA	*Donkey orchid virus A*	JX156422
EAPV	*East Asian pasiflora virus*	AB246773
FreMV	*Freeria mosaic virus*	FM206346
FVY	*Fritillay virus Y*	AM039800
GSMV	*Gloriosa stripe mosaic virus*	EF427894
HarMV	*Hardebergia mosaic virus*	HQ161080
HiMV	*Hippeastrum mosaic virus*	JQ395040
IJMV	*Iranian Johnsongrass mosaic virus*	JQ692088
JYMV	*Japanese yam mosaic virus*	AB027007
KeMV	*Keunjorong mosaic virus*	JF838187
KoMV	*Konjac mosaic virus*	AB219545
LMoV	*Lily mottle virus*	JN127341
LMV	*Lettuce mosaic virus*	AJ306288
LuMV	*Lupin mosaic virus*	EU847625
LYSV	*Leek yellow stripe virus*	HQ258895
MDMV	*Maize dwarf mosaic virus*	AJ001691
MWMV	*Moroccan watermelon mosaic virus*	EF579955
NDV	*Narcissus degeneration virus*	AM182028
NLSYV	*Narcissus late season yellow virus*	JQ326210
OrMV	*Ornithogalum mosaic virus*	JQ807995
OYDV	*Onion yellow dwarf virus*	AB219833
PanVY	*Panax virus Y*	GQ916624
PeMoV	*Peanut mottle virus*	AF023848
PenMV	*Pennisetum mosaic virus*	AY642590
PepMoV	*Pepper mottle virus*	AB126033
PepSMV	*Pepper severe mosaic virus*	AM181350
PepYMV	*Pepper yellow mosaic virus*	AB541985
PkMV	*Pokeweed mosaic virus*	JQ609095
PLDMV	*Papaya leaf distortion mosaic virus*	AB088221
PPV	*Plum pox virus*	EF569215
PRSV	*Papaya ringspot virus*	AB369277
PSbMV	*Pea seed-borne mosaic virus*	AJ252242
PTMV	*Peru tomato mosaic virus*	AJ437280
PVA	*Potato virus A*	AF543212

PVB	*Potato virus B*	JX294310
PVMV	*Pepper veinal mottle virus*	DQ645484
PVY	*Potato virus Y*	AB185833
PWV	*Passion fruit woodiness virus*	AB761400
ScMV	*Scallion mosaic virus*	AJ316084
SMV	*Soybean mosaic virus*	EU871724
SPFMV	*Sweet potato feathery mottle virus*	KU511268
SPLV	*Sweet potato latent virus*	KC443039
SPV2	*Sweet potato virus 2*	JN613807
SPVC	*Sweet potato virus C*	GU207957
SPVG	*Sweet potato virus G*	JN613805
SrMV	*Sorghum mosaic virus*	U57358
SuCMoV	*Sunflower chlorotic mottle virus*	GU181199
SuCMV	*Sugarcane mosaic virus*	AF494510
SuMMV	*Sunflower mild mosaic virus*	JQ350738
TelMV	*Telosma mosaic virus*	DQ851493
TEV	*Tobacco etch virus*	DQ986288
TFMV	*Thunberg fritillary mosaic virus*	AJ851866
ToNSV	*Tomato necrotic stunt virus*	JQ314463
TuMV	*Turnip mosaic virus*	AB093596
TVBMV	*Tobacco vein banding mosaic virus*	EF219408
TVMV	*Tobacco vein mottle virus*	X04083
VSV	*Vallota speciosa virus*	NC017977
VVY	*Verbena virus Y*	EU564817
WMV	*Watermelon mosaic virus*	AB218280
WPMV	*Wild potato mosaic virus*	AJ437279
WTMV	*Wild tomato mosaic virus*	DQ851495
WVMV	*Wisteria vein mosaic virus*	AY656816
YBMV	*Yambean mosaic virus*	JN190431
YMMV	*Yam mild mosaic virus*	JX470965
ZaMMV	*Zantedeschia mild mosaic virus*	AY626825
ZYMV	*Zucchini yellow mosaic virus*	AY188994

Supplementary Table 2. List of primers used in the study

Name	Sequence 5´-> 3´
2672	GGGATCTCCTTTGCCCCAG
2673	CACAACCTGATTTAAATCAGAGTAGTGGATTATCTCATTGCTC
2674	GATTTAAATCAGGTTGTGGTTCACCAAGCTGACGAAGGTCTTGAAGTGGATAAGTG
2675	GGGTATGAATACTCCGAAAG
2676	AATGGCAACTGTAATGGC
2678	GCCCAAGTTTCATTCGTGC
2709	GAATGTCGTAGTACATCAGGCCATGGCAACTGTAATGGCATCTGCG
2679	AAGCTCTACTATGGAAAGATTTC
2680	GCCTAGCCTACTATGTAGTG
P1sF	CACCATGGCAACTGTAATGGC
HCsR	CTAGCCTACTATGTAGTGTTTCAT
HCsmutF	TCTTTTGCGAATGCGATATCACAGAAATCATC
HCsmutR	TATCGCATTCGCAAAAGATTGTAGTGAACC
697	GGGGACAAGTTTGTACAAAAAAGCAGGCTGGAAAATATAAAAACTCAACACAAC
354	GGACTAGTGAATTCGTCGATTCG
3227	CAACTTCAAGACCCGCCACA
3228	TCTGGTAAAAGGACAGGGCCA
2806	GACCCTGATGTTGATGTTCGCT
2807	GAGGGATTTGAAGAGAGATTTC
630	ACRCACAATCCCACTATCC
270	CAGAAACTCGGAATGC
2508	GGGGGTCCTAGCGCGAGTC
441	TCCTGCAGATAACTTTTTTCAACC
90	CGGACCCAATGCAAG
317	TGAACCACTATTGAACAG

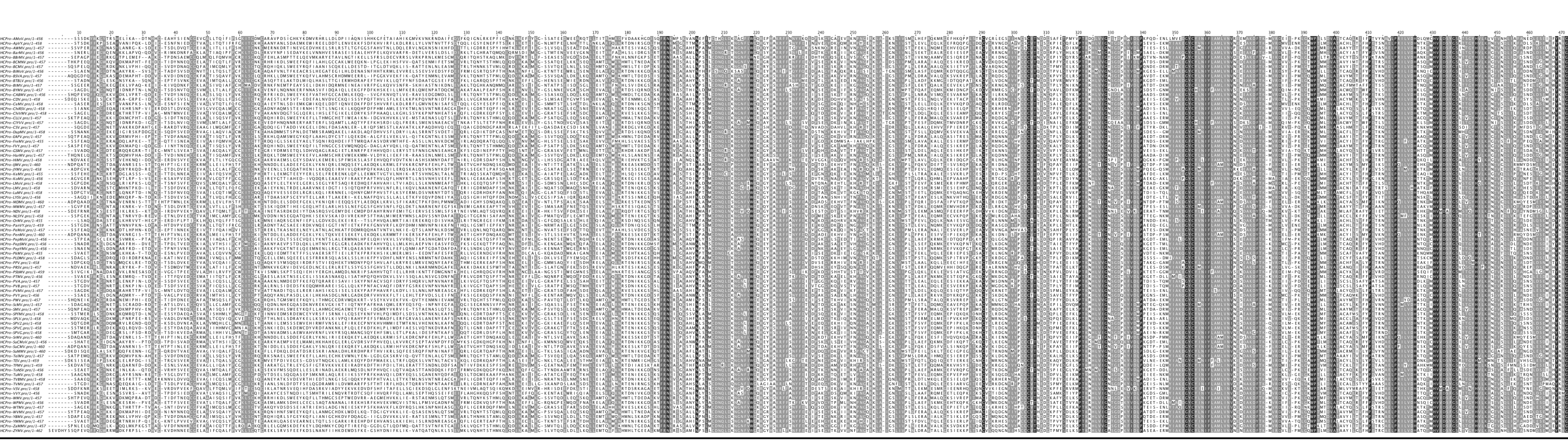

Figure S1. Sequence alignment of potyviral HCPro. Amino acid sequences of HCPro from 86 different potyviral species have been used for the alignment. Positions in which amino acids are identical are marked in black. Dark grey marks positions in which amino acids are not identical, but are conserved [15] in all 86 species. Pale grey marks positions in which amino acids are conserved at least in 70 viral species (~80% of the total).

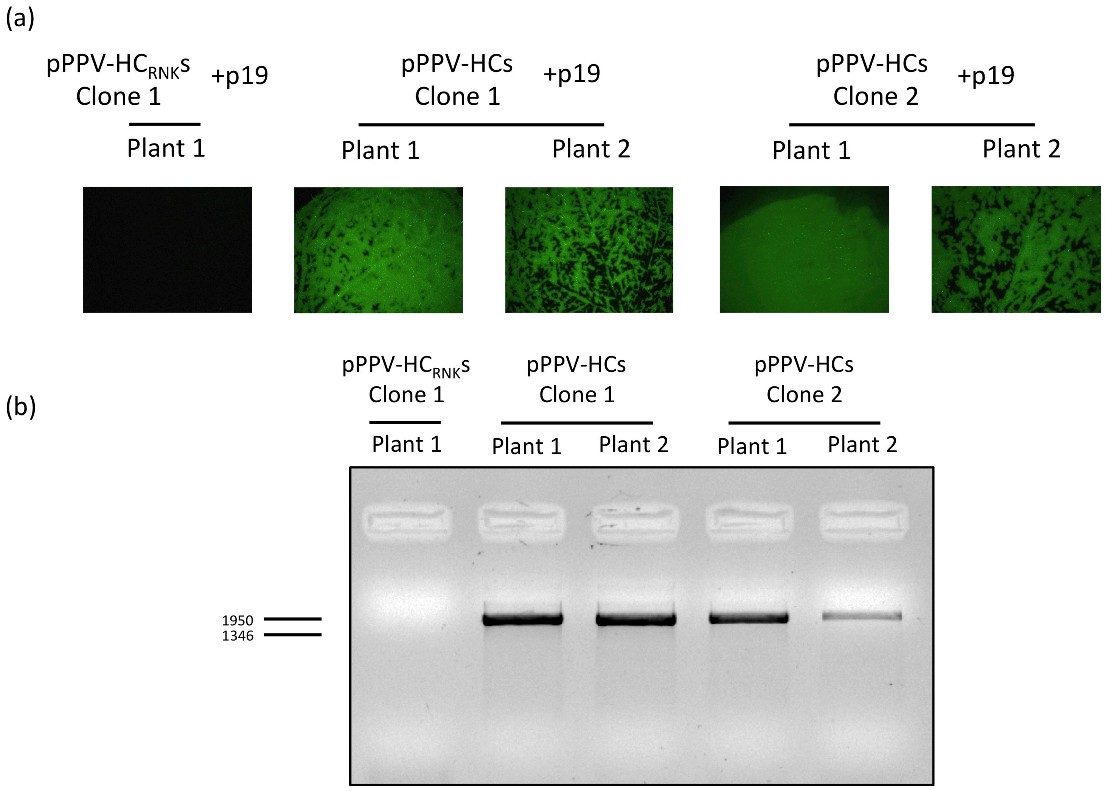

Figure S2. Second experiment of the infection of *N. benthamiana* plants with a chimeric PPV carrying the coding sequence of SPFMV HCPro instead of the PPV HCPro cistron. (**a**) Pictures of agroinfiltrated leaves of *N. benthamiana* plants taken under an epifluorescence microscope at 14 dpa. (**b**) PCR amplification after reverse transcription using primers for specific amplification of the region between P1 and P3. The size (in bp) of DNA markers run in the same gel is shown to the left of the panel.

Full-length gel, Figure 4

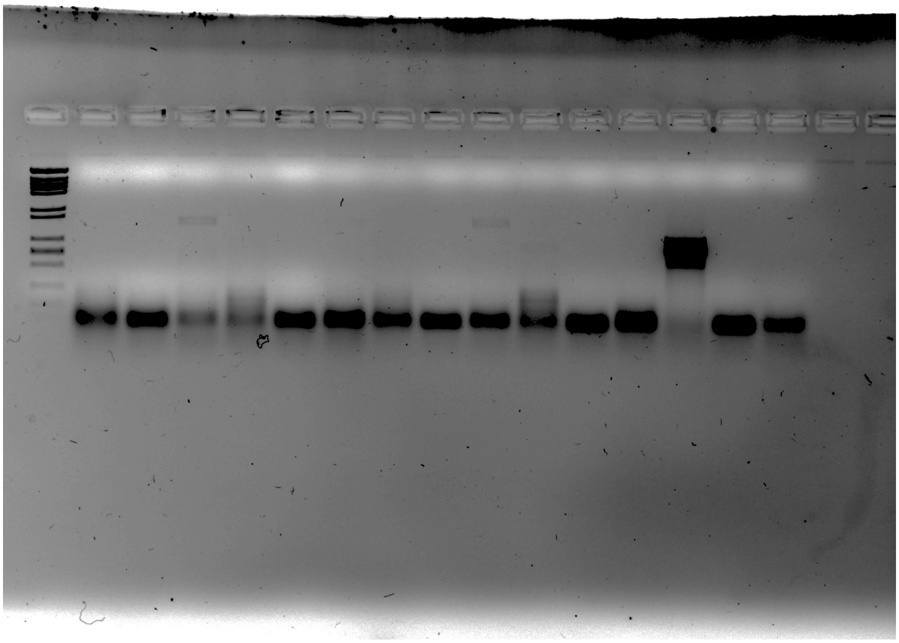

Full-length blots, Figure 5

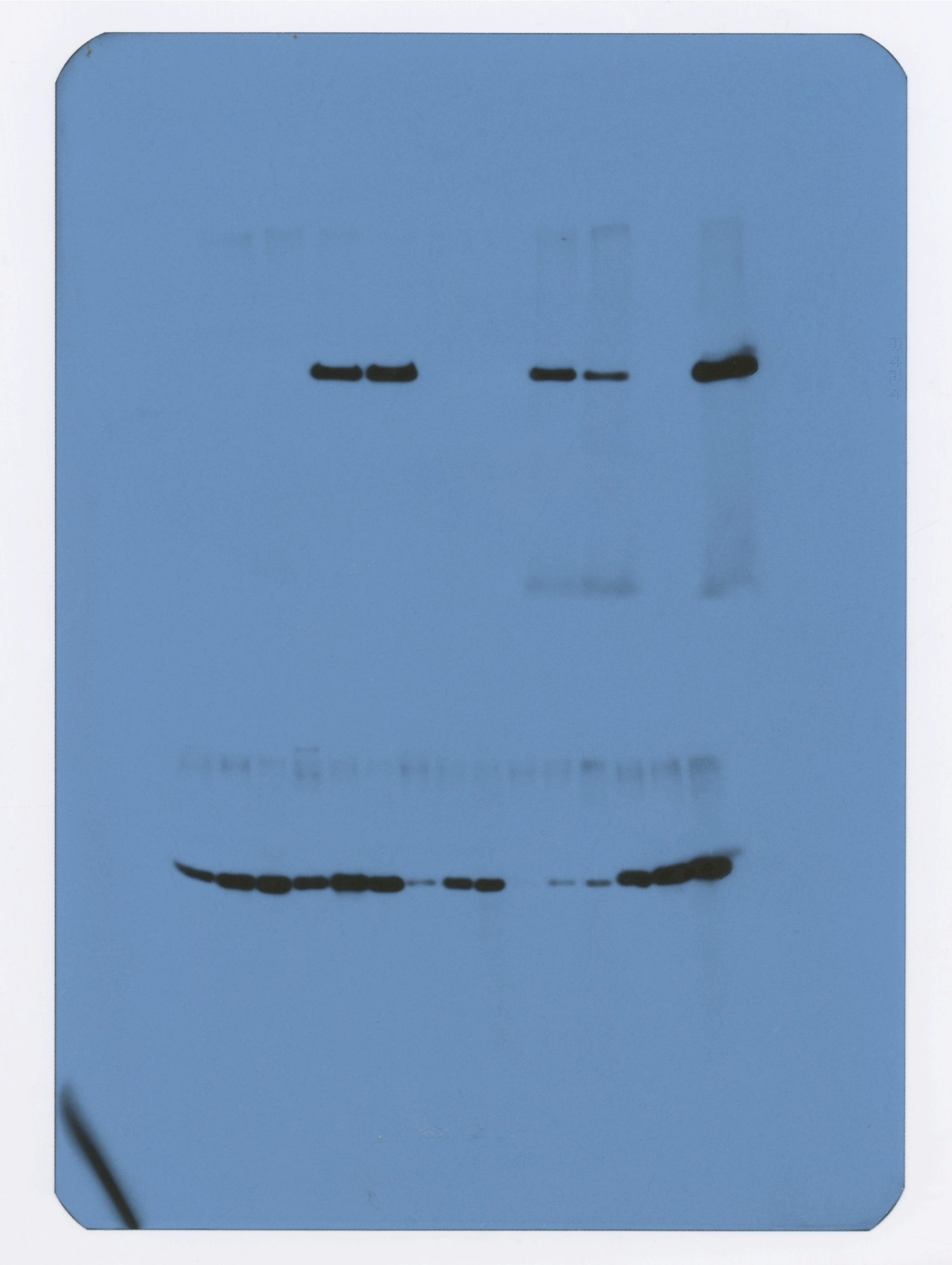

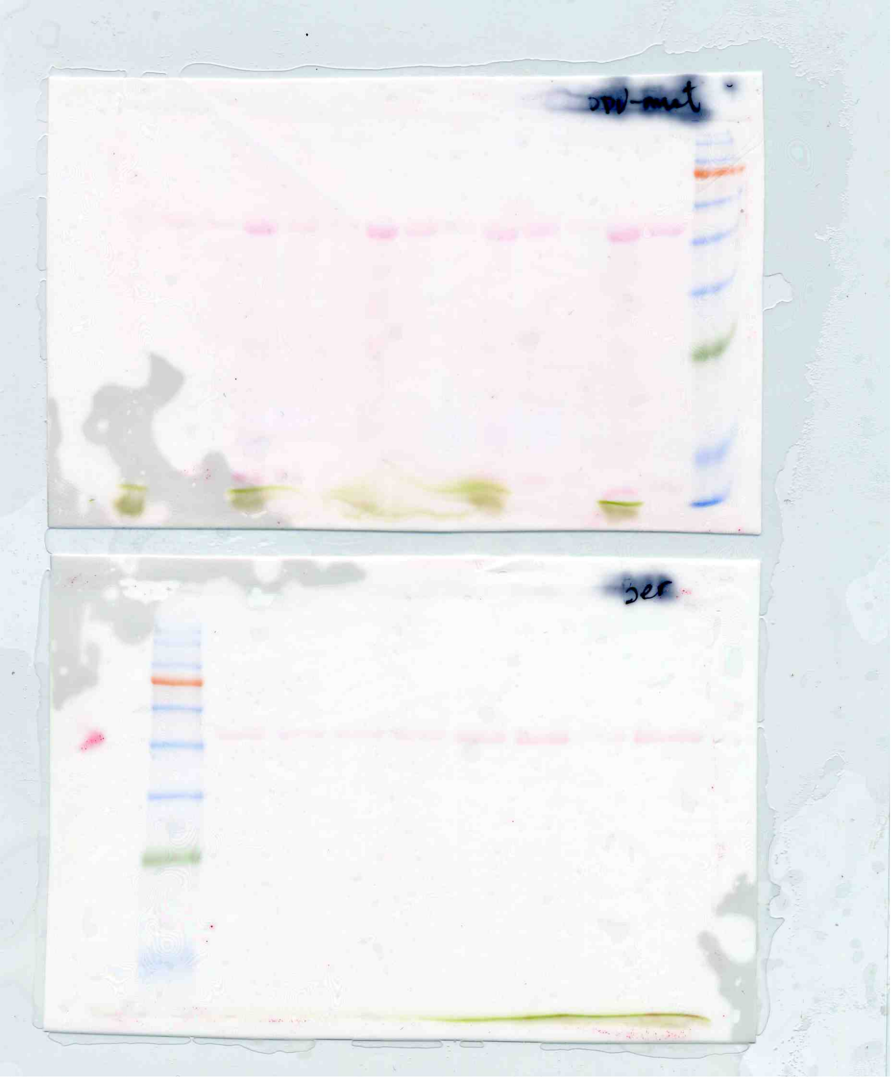

Full-length gel, Figure S2

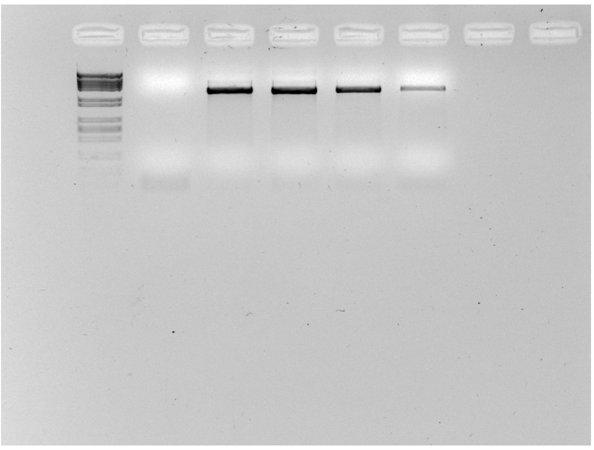

Supplement: Supplementary file 1 — Supplementary File [file 41598_2018_34358_MOESM1_ESM.pdf]
